# Editorial Comment: Increased risk of dementia among patients with overactive bladder treated with an anticholinergic medication compared to a beta-3 agonist: a population-based cohort study

**DOI:** 10.1590/S1677-5538.IBJU.2021.04.02

**Published:** 2021-07-20

**Authors:** Jorge Moreno-Palacios

**Affiliations:** 1 IMSS Servicio Urología UMAE Hospital de Especialidades CMN Siglo XXI Ciudad de México México Servicio Urología UMAE Hospital de Especialidades CMN Siglo XXI, IMSS, Ciudad de México.

Blayne Welk ^1^^,^ ^2^^,^ ^3^, Eric McArthur ^2^

BJU Int. 2020 Jul;126(1):183-190

## COMMENT

There is an increase in the number of publications on the effect of the drugs we use for the treatment of lower urinary tract symptoms on the central nervous system, especially those related to the treatment of overactive bladder (OAB), studies on anticholinergics in general have shown that users are at higher risk of new-onset dementia (1, 2). However beta 3 agonists were recently introduced as a new class of medical therapy for OAB. In this study the objective was to determine if there was an increased risk of new-onset dementia among first-time users of OAB anticholinergics compared to beta-3 agonists in a retrospective, matched-cohort study. They match more 40,324 vs 23,662 new anticholinergic and beta 3 agonists users respectively. The median of prescription for each group was 30 vs 64 days. There was an increased risk of dementia among anticholinergic users compared to beta-3 agonist users (hazard ratio 1.23, 95% confidence interval 1.12– 1.35). Interestingly in the stratified analysis there was a significant effect modification based on both gender and age; men and those aged ≤ 75 years on anticholinergics had the highest risk of dementia relative to similar beta-3 agonist users. The authors didn't find a significant differential risk of dementia based on the type of anticholinergic medication used.

Unlike other studies whose design were case-control, this is a cohort where a greater causal association can be found and the hazard ratio was calculated. In addition, this study analyzed anticholinergics used for OAB, prior research has relied largely on complex calculations using anticholinergic burden scales to account for all potential medications. However, it is a study based in prescriptions without knowing the adherence to treatment that we know is low in this disease, especially with antimuscarinics. Further research should be carried out to explore the identified effect modifiers of gender and age in this patient population, and to assess the differential effects of specific OAB anticholinergics.

The present study supports a small but measurable increased risk in dementia diagnosis with anticholinergic medications, as urologists we must be aware of this association more in the setting of elderly patients that are exposed to polypharmacy.
